# Supplementary material for: DFT Investigations of the Vibrational Spectra and Translational Modes of Ice II
Source: Molecules. 2019 Aug 28;24(17):3135. doi: 10.3390/molecules24173135 (PMC6749557; doi:10.3390/molecules24173135)
Supplement: Supplementary file 1 [file molecules-24-03135-s001.zip › molecules-586867-SM -proofed/S1-4/S1.pdf]

Normal modes (0.3Gpa)	Normal modes (0.5Gpa)	Normal modes (1.0Gpa)	IR[22, 23]	Raman[24, 25]
50(2)	54(2)	77(2)		
78(2)	82(2)	93		
84	86	103(2)		
86(2)	90(2)	104(2)		72
91	93	110		
108	115	123		
111(2)	117	132		104
116	118(2)	135(2)		
124(2)	130(2)	142(2)	107	
129	135	146		
137	142	154		
147(2)	154(2)	173(2)		152
149	157	182	136	
157(2)	164(2)	183(2)	151	
167	176	194		187
197(2)	206(2)	228(2)	186	
210	219	243(2)		200
216(2)	223(2)	246	253	
254(2)	262(2)	281(2)		262
285	293	313	295	
289(2)	298(2)	323(2)		268
307	**318**	346	335	322
515(2)	522(2)	539(2)		489
516(2)	523(2)	540(2)	473	
			483	
			498	
518	527	548	516	
531	538	554		495
585	597	618	533	
590(2)	599(2)	621(2)		573
598	610	635		597
602	611	639	544	
620(2)	630(2)	654(2)	593	
641	654	679(2)		617
644(2)	655(2)	681	642	
647(2)	657(2)	682(2)		648
651	660	683	660	
721	733	751	700	
741	752	779		685
765(2)	774(2)	794(2)		715
791(2)	804(2)	835(2)		775
802(2)	815(2)	845(2)	745	

834(2)	844(2)	868(2)	800	
842(2)	853(2)	879(2)		845
860	871	898	835	
871(2)	881(2)	907(2)	960	
899	909	934		950
959	969	994		
			1066	
1659(2)	1659(2)	1658(2)		
1660	1661	1662		
1664(2)	1665(2)	1666(2)		
1679	1680	1682	1690	
1685(2)	1686(2)	1689(2)		
1702	1703	1706		
1707	1708	1713		
1721(2)	1723(2)	1729(2)	1748	
			2220	
			2300	
**3205**	**3193**	**3158**		**3189**
3210	3198	3164		3225
3239(2)	3227(2)	3194(2)	3000	
3257(2)	3245(2)	3214(2)	3225	
3291(2)	3279(2)	3247(2)		
3315	3304	3273	3280	
3329(2)	3321(2)	3295(2)		3270
3345	3335	3308	3390	
3410	3393	3352		3306
3420(2)	3405(2)	3372(2)		3340
3424	3412	3383	3470	
3438(2)	3425(2)	3393(2)		
3470	3456	3428		
3479(2)	3466(2)	3438(2)		3400
3482(2)	3469(2)	3439(2)	3500	
3495	3483	3455		3465
